# Feasibility of quantitative susceptibility mapping (QSM) of the human kidney

**DOI:** 10.1007/s10334-020-00895-9

**Published:** 2020-11-24

**Authors:** Eric Bechler, Julia Stabinska, Thomas Thiel, Jonas Jasse, Romans Zukovs, Birte Valentin, Hans-Jörg Wittsack, Alexandra Ljimani

**Affiliations:** 1 Department of Diagnostic and Interventional Radiology, Medical Faculty, Heinrich Heine University Düsseldorf, Moorenstr. 5, 40225 Düsseldorf, Germany; 2 Department of Haematology, Oncology and Clinical Immunology, Medical Faculty, Heinrich Heine University Düsseldorf, Düsseldorf, Germany

**Keywords:** Quantitative susceptibility mapping, Renal MRI, Functional renal imaging

## Abstract

**Objective:**

To evaluate the feasibility of in-vivo quantitative susceptibility mapping (QSM) of the human kidney.

**Methods:**

An axial single-breath-hold 3D multi-echo sequence (acquisition time 33 s) was completed on a 3 T-MRI-scanner (Magnetom Prisma, Siemens Healthineers, Erlangen, Germany) in 19 healthy volunteers. Graph-cut-based unwrapping combined with the T_2_*-IDEAL approach was performed to remove the chemical shift of fat and to quantify QSM of the upper abdomen. Mean susceptibility values of the entire, renal cortex and medulla in both kidneys and the liver were determined and compared. Five subjects were measured twice to examine the reproducibility. One patient with severe renal fibrosis was included in the study to evaluate the potential clinical relevance of QSM.

**Results:**

QSM was successful in 17 volunteers and the patient with renal fibrosis. Anatomical structures in the abdomen were clearly distinguishable by QSM and the susceptibility values obtained in the liver were comparable to those found in the literature. The results showed a good reproducibility. Besides, the mean renal QSM values obtained in healthy volunteers (0.04 ± 0.07 ppm for the right and − 0.06 ± 0.19 ppm for the left kidney) were substantially higher than that measured in the investigated fibrotic kidney (− 0.43 ± − 0.02 ppm).

**Conclusion:**

QSM of the human kidney could be a promising approach for the assessment of information about microscopic renal tissue structure. Therefore, it might further improve functional renal MR imaging.

**Electronic supplementary material:**

The online version of this article (10.1007/s10334-020-00895-9) contains supplementary material, which is available to authorized users.

## Introduction

In recent years, there has been an increasing research interest in functional renal MRI. Several previous studies have demonstrated great potential of MRI biomarkers for characterizing different pathological processes involved in the progression of chronic kidney disease (CKD) [1–3]. A histological hallmark of CKD, and a major cause of progressive renal function loss is the renal interstitial fibrosis. Therefore, interstitial fibrosis degree in the renal tissue is an important indicator in the determination of the reversibility of kidney damage. Up to now, the only reliable clinical tool to evaluate the degree of tubulointerstitial fibrosis is the renal biopsy. Since this diagnostic procedure is invasive, impaired by sampling bias and not arbitrarily repeatable [4, 5], a non-invasive imaging modality able to accurately assess the degree of renal interstitial fibrosis is highly desirable.

Quantitative susceptibility mapping (QSM) is a novel MRI technique, which uses phase images to produce a high structural contrast and quantitative information of the magnetic susceptibility of the tissue [6–9]. In previous studies, QSM has been shown to be sensitive to changes in tissue microstructure or chemical composition [10–12] and is, therefore, a promising, non-invasive approach for the assessment of renal interstitial fibrosis [13].

So far, QSM has mostly been applied to measure pathologic deposits in basal ganglia in various neurological diseases, or as an imaging biomarker of hepatic iron overload [14–17]. More recent studies in animal models have explored the potential of QSM to assess renal microstructure [13, 18, 19]. In particular, Xie et al. [13] demonstrated the sensitivity of QSM in detecting pathology caused by renal inflammation and fibrosis in mice. However, to the best of our knowledge, there has been no adequate study performed in vivo to map susceptibility in the human kidney.

Abdominal QSM is considered technically challenging. First, respiratory movement of the upper abdominal organs leads to limited structural contrast and underestimated susceptibility values [17]. Second, the presence of abdominal fat negatively impacts the estimation of the B0 field map, which is a critical step in the QSM algorithm [17, 20]. Third, the large susceptibility variations around the air–tissue interfaces cause severe streaking artifacts, and thus erroneous QSM maps [21]. Furthermore, as shown in a previous preliminary simulation study by our study group [22], the accuracy of the abdominal susceptibility map is strongly affected by the phase processing step, including unwrapping and background field removal.

The aim of the present work was to evaluate the feasibility of performing in vivo QSM of the human kidney on a clinical MRI system. For this purpose, an optimized MRI acquisition protocol and QSM processing pipeline were employed to obtain kidney QSM maps.

## Methods

### Study population

The study was approved by the local ethics committee, and written informed consent was obtained from all subjects.

Nineteen healthy volunteers (mean age 28.1 ± 12.9 years) without any history of kidney disease or any known systemic disease potentially involving the kidneys participated in the study. Five subjects were measured twice with a time interval of 10 min between measurements and re-positioning in the MRI to evaluate the reproducibility.

Furthermore, a 78-year-old, male patient with severe kidney fibrosis due to a long anamnesis of renal insufficiency (CKD V (eGFR < 15 ml/min/1.73 m^2^) for 25 years, state after kidney transplantation 20 years ago, chronic graft failure and dialysis for the last 5 years) was exemplarily included in the study to evaluate the potential clinical relevance of QSM.

No specific preparations were undertaken prior to the examination [1].

### Data acquisition

**Fig. 1:**
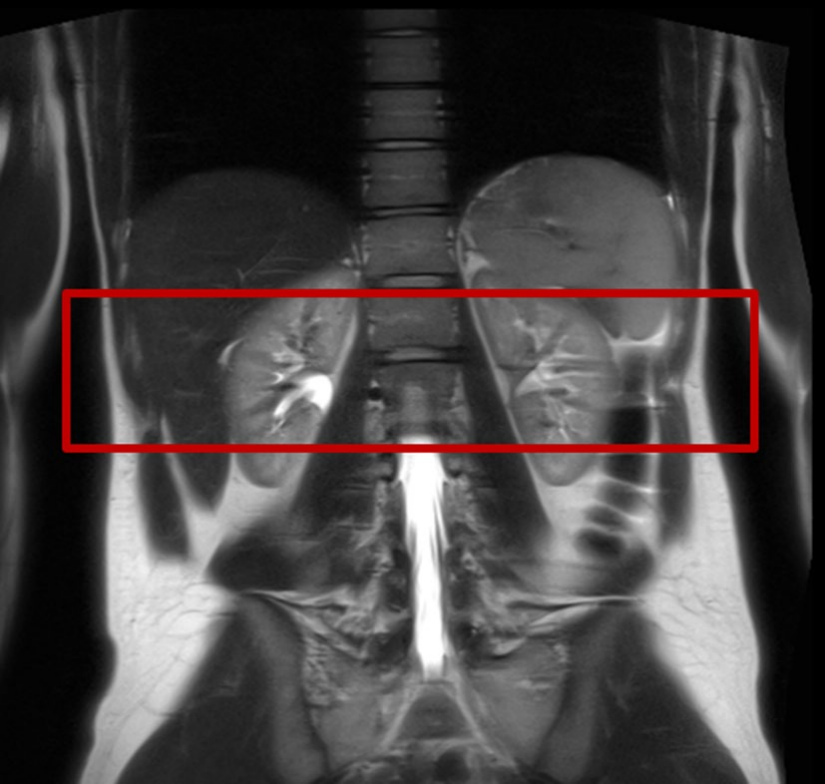
Example of FOV placement for renal QSM acquisition. FOV was placed central in the kidneys to ensure uniform imaging conditions

Data acquisition was performed on a 3 T scanner (Magnetom Prisma, Siemens AG, Healthineers, Erlangen, Germany) using a 32 channel spine coil in combination with a 30 channel body coil. A half-Fourier single-shot turbo spin echo (HASTE) sequence in all three image axes (axial, coronal and sagittal) was used to acquire anatomical images. These anatomical images were used for FOV placement for the following QSM sequence. The FOV was placed central in the kidneys (Fig. 1).

QSM data were acquired using an axial single-breath-hold 3D multi-echo gradient echo sequence with the following parameters: number of echoes = 4; TE_1_/ΔTE/TR = 3.1/3.7/17 ms; flip angle = 15°; acquisition matrix = 256 × 192 × 26; voxel size = 1.64 × 1.64 × 3 mm^3^; bandwidth = 1775 Hz/pixel; slice and phase Fourier encoding = 6/8; parallel imaging acceleration factor = 2; acquisition time 33 s. The settings for renal QSM acquisition were determined in pre-tests to achieve the optimal image quality in the shortest possible acquisition time.

The quality of the breath-hold during the QSM acquisition was verified through visual control by the integrated patient observation camera. Furthermore, the quality of the acquired data was verified by two experienced radiologists in abdominal imaging (A.L. 10 years, B.V. 4 years) before post-processing. In the case of significant motion artifacts in the data, the QSM acquisition was repeated immediately or excluded from further analysis if the repetition failed.

### Post-processing

**Fig. 2:**
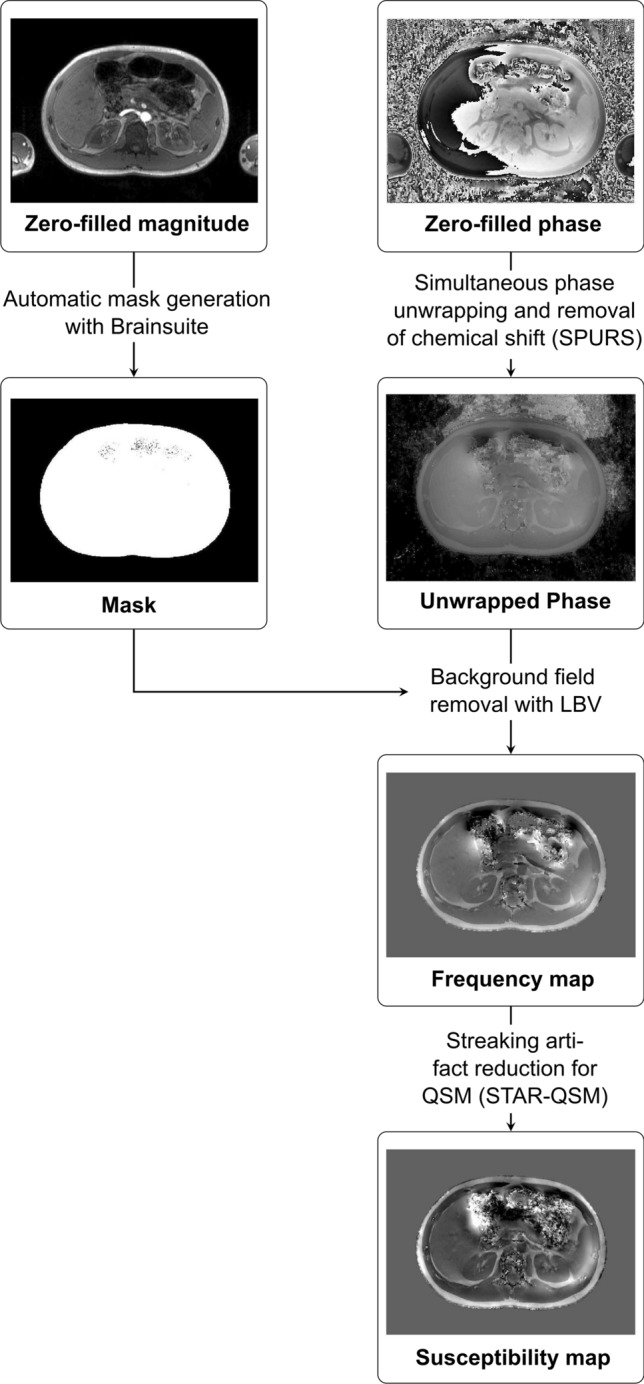
Flow chart displaying the reconstructions steps the calculation of the susceptibility maps. The starting point of the reconstruction is the zero-filled magnitude and phase data. Simultaneous phase unwrapping and removal of chemical shift (SPURS) was applied to zero-filled phase data to remove chemical shift effects between water and fat. Further, a T2*-IDEAL approach was applied to calculate the resulting fat-corrected field maps (unwrapped phase). Masks of the whole abdomen were automatically generated from zero-filled magnitude data for background field removal with the help of the Laplacian boundary value (LBV) algorithm. In a final step, the ill-posed inverse problem was solved by the streaking artifact reduction for QSM (STAR-QSM) method from the STI-Suite resulting in susceptibility maps

The flow chart in Fig. 2 displays the reconstruction steps that were undertaken to estimate the kidney QSM maps. All calculations were performed using MATLAB (R2018a; The MathWorks, Inc., Natick, MA).

In the first step, the data were zero-filled leading to a voxel size of 0.8 × 0.8 × 2.25 mm^3^. The post-processing for QSM was originally optimized for brain imaging, where the contribution of fat to the MRI signal is minimal [20]. However, applications outside the brain especially in the abdomen require effective fat removal to avoid quantification bias in the susceptibility maps. In this study, the unwanted chemical shift effect between water and fat was eliminated by a method called *Simultaneous phase unwrapping and removal of chemical shift *(*SPURS*) [20]. SPURS uses a graph-cut-based unwrapping to eliminate the phase wraps in the zero-filled phase data. Further, a T_2_*-IDEAL approach [23] was applied to calculate the resulting fat-corrected field maps, which were used as an input for the background field removal.

In this study, masks of the whole abdomen were automatically generated on the axial images (Brain Surface Extractor (BSE) from BrainSuite, Version 18a, University of California) from the zero-filled magnitude data to remove the unwanted air outside of the abdomen. After visual quality control of the segmentation, the generated masks were used to remove the background field with the help of the *Laplacian boundary value* (*LBV*) algorithm [24], which is part of the MEDI-toolbox [25].

In a final step, the ill-posed inverse problem was solved by the streaking artifact reduction for QSM (STAR-QSM) method [26] from the STI-Suite [9] resulting in susceptibility maps. Both LBV and STAR-QSM were run with default settings.

**Fig. 3:**
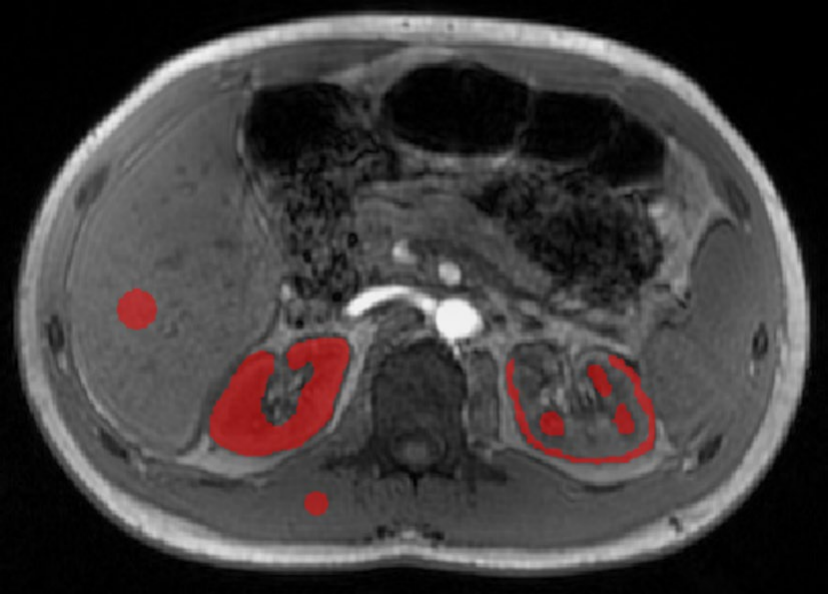
Example of ROI placement. Magnitude image of the abdomen with exemplary regions of interest (ROIs) drawn in the paravertebral muscle tissue (336 pixels), liver (900 pixels), the entire kidney (5533 ± 1792 pixels and 4756 ± 1142 pixels for left and right kidneys, respectively), renal cortex (1260 ± 279 pixels and 1245 ± 265 pixels for left and right kidneys, respectively) and renal medulla (993 ± 293 pixels and 962 ± 392 pixels for left and right kidneys, respectively)

The software ITK-SNAP (version 3.8.0, University of Pennsylvania) was used to manually draw regions of interest (ROIs) in the paravertebral muscle tissue (336 pixels), liver (900 pixels), the entire kidney (5533 ± 1792 pixels and 4756 ± 1142 pixels for left and right kidneys, respectively), renal cortex (1260 ± 279 pixels and 1245 ± 265 pixels for left and right kidneys, respectively) and renal medulla (993 ± 293 pixels and 962 ± 392 pixels for left and right kidneys, respectively) (Fig. 3). All ROIs were drawn over three consecutive slices and the mean susceptibility and standard deviation (SD) was calculated for each organ and subject. The paravertebral muscle tissue was used as a reference for QSM quantification in the current study to ensure consistency of the susceptibility values (Supplement Material, Table S1) [27].

To examine whether global post-processing effects bias the calculated QSM values, e.g., non-local errors from unreliable field estimation across the ROIs, QSM values in the liver were determined and compared to the literature values available.

Furthermore, accuracy of outside air removal is an important step for QSM quantification. To evaluate the influence of the mask definition quality on the QSM values, the mask definition was varied in one healthy subject. Thus, four different masks (without air outside of the abdomen, containing a small and a large amount of outside air and containing whole image area) were applied to the dataset, respectively, and susceptibility values of the right kidney were compared.

### Statistical analysis

The susceptibility values of the left and right kidney, as well as the cortex and medulla in both kidneys, of the healthy control group were averaged across all subjects and compared to the mean and SD inside the right fibrotic kidney to assess an example of the potential clinical relevance of QSM.

Furthermore, Wilcoxon test was used to compare the QSM results of the left and right kidneys as well as renal cortex and medulla. Besides, a Pearson correlation between the kidney and liver susceptibilities was calculated for the healthy control group.

## Results

**Fig. 4:**
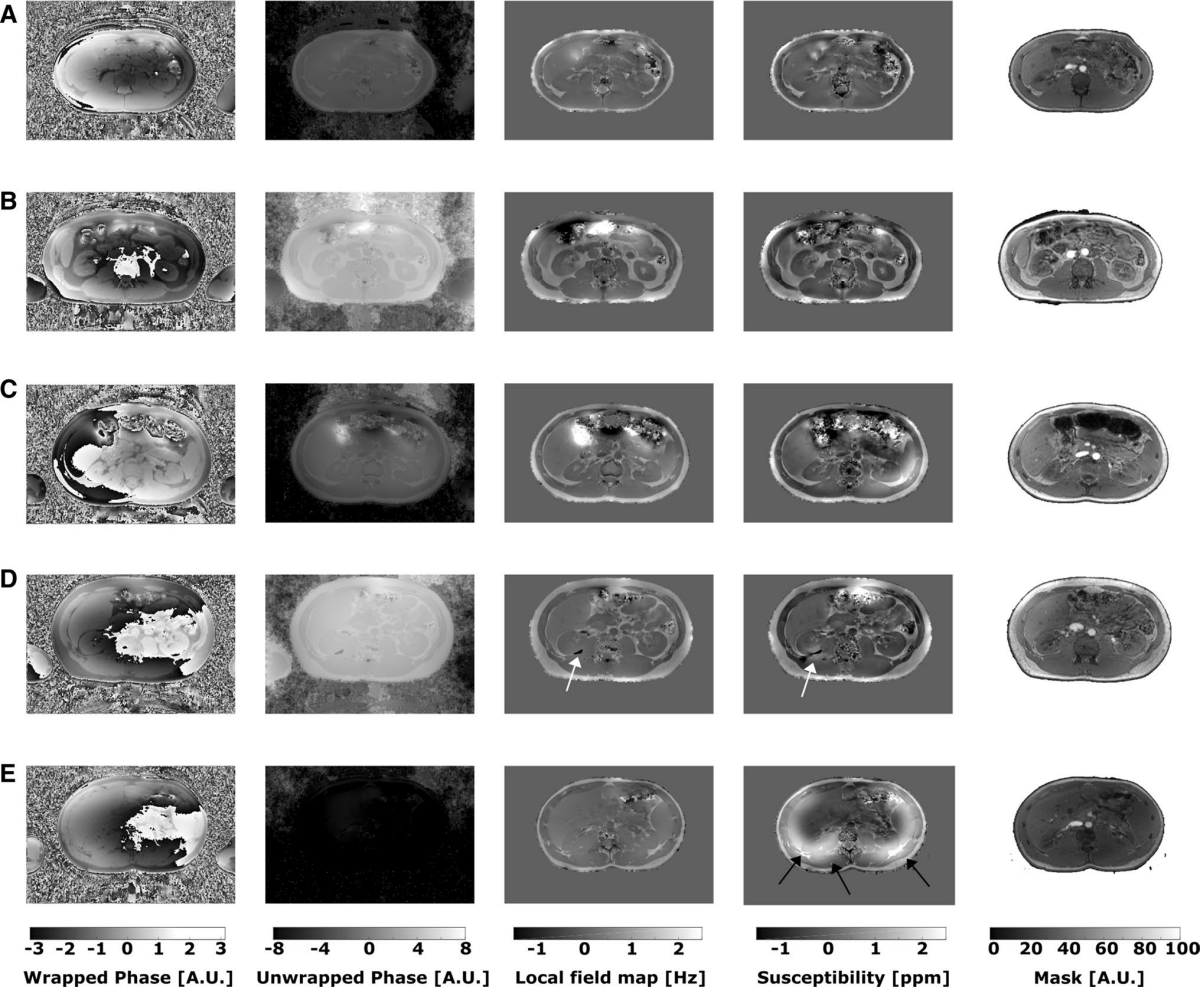
Examples of wrapped and unwrapped phase images as well as local field and susceptibility maps and the corresponding mask of the upper abdomen of five healthy volunteers. Anatomical structures are clearly distinguishable and only a few artifacts are present in the intestinal area (**a**–**c**). In one case, phase unwrapping failed close to the kidney, leading to inaccurate susceptibility values (**d**, white arrow). Example of severe artifacts due to air in the lungs (**e**, black arrows), which were present in two healthy volunteers. Both datasets were removed from further post-processing

Two healthy subjects were excluded from further analysis due to severe artifacts at the boundary between the lungs and surrounding tissue (Fig. 4e).

**Fig. 5:**
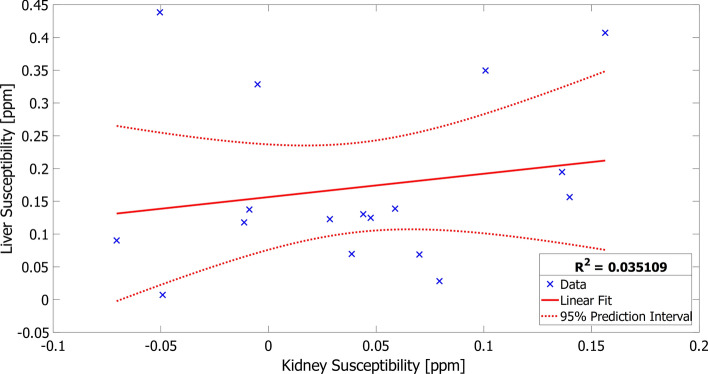
Pearson correlation plot between renal and hepatic susceptibility values of the healthy volunteers. No correlation was observable between renal and hepatic QSM values (*R*^2^ = 0.035), indicating that the global post-processing effects did not affect the QSM quantification

QSM was successfully quantified in the 17 remaining healthy volunteers and the patient with renal fibrosis (Supplement Material, Figs. S1–S3). The parenchymatous upper abdominal organs, as liver and kidney, were clearly distinguishable in these datasets (Fig. 4). In one case, phase unwrapping failed in a small area close to the kidney, leading to inaccurate susceptibility values in that region (Fig. 4d). However, only a small part of the kidney was affected and was considered during the ROI placement. No correlation was observable between the QSM values of the kidney and the liver (*R*^2^ = 0.035) (Fig. 5), indicating that no global effects from post-processing bias the calculated susceptibility values.

**Fig. 6:**
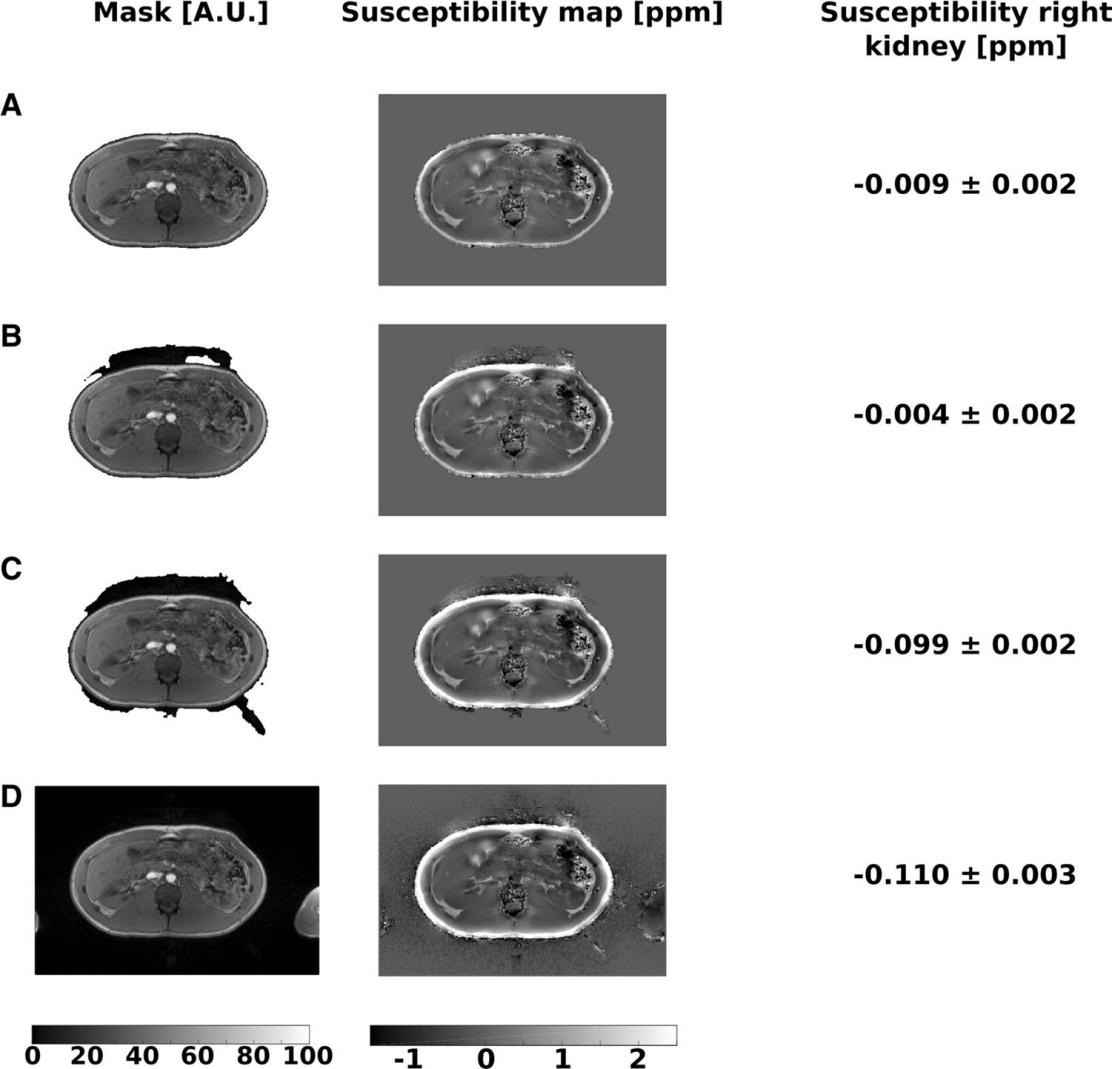
Examples of susceptibility maps and values for the variation of mask definition for outside air removal in one healthy volunteer. No changes of QSM values of the right kidney could be identified in the case of a relatively small segmentation inaccuracy with no outside air left after segmentation (**a**) and containing a small amount of outside air (**b**). However, larger amounts of outside air led to inaccurate susceptibility values (**c**, **d**)

Figure 6 displays the susceptibility maps and values for the variation of mask definition in one healthy volunteer. No changes of renal QSM values could be identified in the case of a relatively small segmentation inaccuracy (Fig. 6a, b). However, larger amounts of air led to inaccurate susceptibility values (Fig. 6c, d).

The mean renal susceptibility values of the healthy volunteers were 0.04 ± 0.07 ppm (range − 0.07 to 0.16 ppm) for the right kidney and − 0.06 ± 0.19 ppm (range − 0.35 to 0.39 ppm) for the left kidney, respectively (Table 1). The mean susceptibility values of the right and left kidneys were significantly different (*p* < 0.05) showing a wider range of values for the left kidney (Table 1). No significant difference between cortical and medullary QSM values of the right or the left kidney could be determined (*p* > 0.05).

**Table 1:** Susceptibility values of the whole kidney, the cortex and the medulla of healthy volunteers, averaged over all 17 subjects

Region	Susceptibility (ppm)
Kidney right	0.04 ± 0.07
Cortex right	0.02 ± 0.08
Medulla right	0.06 ± 0.08
Kidney left	− 0.06 ± 0.19
Cortex left	− 0.06 ± 0.20
Medulla left	− 0.03 ± 0.15

Significant difference of QSM values of the left and the right kidneys (*p* < 0.05) are probably based on higher motion artifacts in the left kidney. Considering the standard deviation, the susceptibility of healthy renal tissue fluctuates around 0 in the current study. No significant difference between cortical and medullar QSM values of the right or the left kidneys could be determinate (*p* > 0.05)

Liver susceptibility values measured in healthy volunteers and the patient with renal fibrosis were in the same range 0.17 ± 0.13 ppm and 0.15 ± 0.01 ppm for healthy volunteers and the patient with renal fibrosis, respectively (Table 2).

**Table 2:** Susceptibility values of the right kidney and the liver of healthy volunteers, averaged over all 17 subjects, and the patient with severe renal fibrosis

Group	Right kidney (ppm)	Liver (ppm)
Healthy volunteers	0.04 ± 0.07	0.17 ± 0.13
Fibrosis	− 0.43 ± − 0.02	0.15 ± 0.01

The QSM value of the right kidney of patient with renal fibrosis is significantly different from the QSM values measured in the right kidney of healthy volunteers. However, liver QSM values measured in healthy volunteers and the patient with renal fibrosis are in the same range, excluding global effects bias renal QSM results

Reproducibility measurements in five subjects revealed a good reproducibility for both liver and the right kidney QSM with no significant difference in hepatic or renal susceptibility values between both measurements (*p* = 0.48) (Table 3). The susceptibility values for the right kidney were 0.02 ± 0.06 ppm and − 0.03 ± 0.11 ppm for the test and re-test measurements, respectively. Liver susceptibility was 0.16 ± 0.10 ppm and 0.12 ± 0.07 ppm, respectively.

**Table 3:** Reproducibility results

Participant	Liver (ppm)	Right kidney (ppm)	
Original	0.16 ± 0.01		0.02 ± 0.06
Reproducibility	0.12 ± 0.07		− 0.03 ± 0.11

Mean ± standard deviation susceptibility values for the right kidney and liver averaged over all five reproducibility subjects. Good reproducibility for both liver and the right kidney QSM with no significant difference in hepatic or renal susceptibility values between both measurements (*p* = 0.48)

**Fig. 7:**
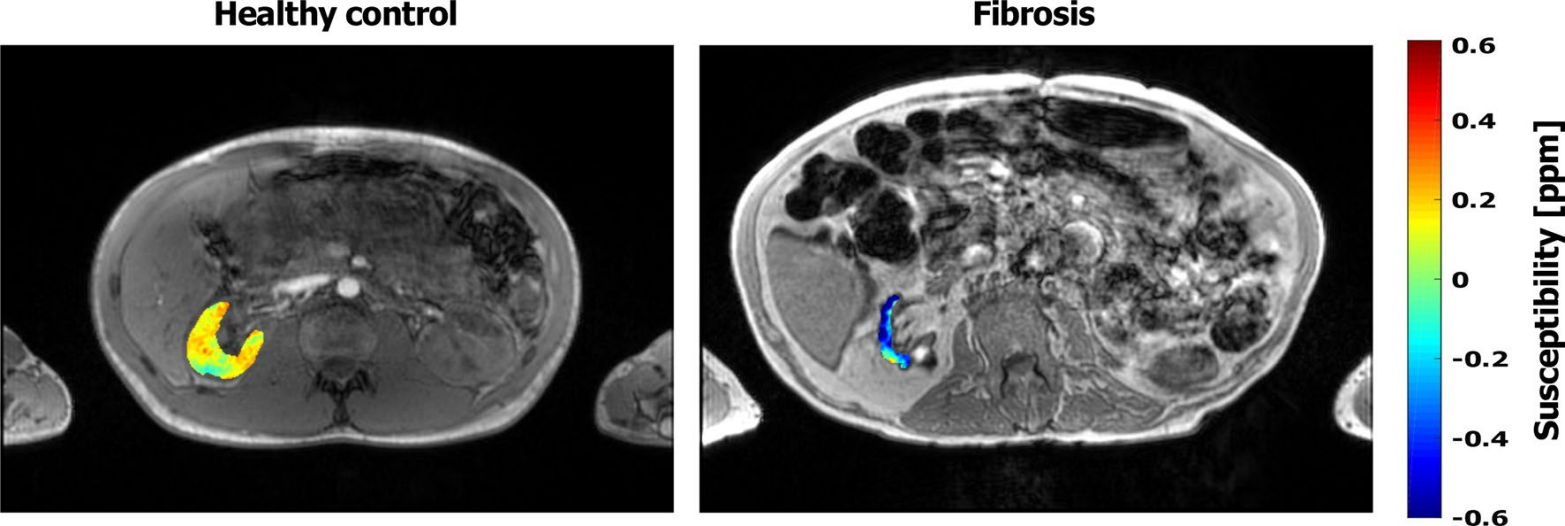
Magnitude images overlaid with the QSM maps of the right kidney for a healthy volunteer (left image) and the patient with renal fibrosis (right image). The fibrotic kidney shows a strong diamagnetic value (− 0.43 ± − 0.02 ppm), which was substantially lower than the QSM value measured in the healthy renal tissue (right kidney 0.04 ± 0.07 ppm)

Figure 7 shows the QSM maps overlaid onto the magnitude images for a healthy volunteer and the one investigated patient with renal interstitial fibrosis. The susceptibility of the right fibrotic kidney was strongly diamagnetic (− 0.43 ± 0.02 ppm).

## Discussion

QSM is a novel, promising approach for the assessment of information about tissue microstructure and function. In this work, we demonstrated the feasibility of performing in vivo QSM of the human kidney on a clinical MRI system. The presented acquisition scheme and further the implemented QSM processing pipeline, composed of the state-of-the-art QSM methods, was successful in 90% of the examined subjects and led to reproducible results. Anatomical structures in the abdomen were clearly distinguishable on the QSM maps and only few artifacts were present in the intestinal area. Besides, the difference between the mean QSM values obtained in the healthy kidneys and the susceptibility of the fibrotic kidney shows the potential of QSM for distinguishing between healthy and pathological renal tissue. As this finding is based on a single fibrotic case only, further studies with larger patient population should prove the diagnostic value of QSM in future.

QSM has already been applied in the human abdomen before [17, 27, 29, 30], showing promising results as a new MRI technique. However, the method poses some technical challenges that have hindered its in vivo application to the human kidney so far. By combining and optimizing already established image post-processing steps for QSM, some of these issues have been resolved successfully in the current study. First, free-breathing-induced motion artifacts were eliminated by acquiring data during a single breath-hold at end-inspiration. Second, the unwanted chemical shift effect between water and fat were removed by employing an advanced post-processing pipeline. Third, the adequate acquisition and post-processing parameters were determined to minimize the artifacts and provide reproducible results.

To the best of our knowledge, this is the first study performing in vivo QSM in the human kidney on a clinical MRI system. Since there are no literature renal QSM values available, we were only be able to compare the susceptibility values of the liver obtained in our study with those reported by the other groups. In the study by Lin et al. [27], the calculated liver susceptibility ranged from 0.23 to 5.94 ppm. However, their study was focused on patients with hepatic iron overload (haemochromatosis). Individuals with less hepatic iron deposits showed QSM values of about 0.34 ppm and were considered healthy. In the study of Dong et al. [20], mean liver susceptibility values of 0.23 ± 0.07 ppm were determined. Overall, the liver QSM values measured in the current study (range 0.01–0.44 ppm) are consistent with the previous research, indicating that no global effects bias our results.

Poor mask definition could have influence on the renal QSM values. Therefore, optimal accuracy of outside air removal is an important step for QSM quantification. In the current study, we varied the mask definition of one healthy subject. In this example, no significant changes of QSM values of the right kidney could be identified in the case of a relatively small segmentation inaccuracy, as it was the case in our study. However, a systematic evaluation of the influence of the mask definition on the QSM values could be an object of further simulation studies.

In this study, the variability of QSM values of the left kidney was significantly higher than those of the right kidney (*p* < 0.05). This difference might be due to higher cardiac artifacts of the left kidney, as shown in several previous renal MRI studies [28]. This effect might be reduced with further development of renal QSM.

Cortico-medullary differentiation was not possible by QSM in the current study (*p* > 0.05), presumably due to the moderate resolution of the method. Improvement of the resolution of QSM to enable cortico-medullar differentiation should be an object of further studies.

To examine the potential diagnostic value of QSM, a patient with renal fibrosis as an example of an end-stage renal pathology was included in the study. Previously, it has been reported that renal fibrosis increases the diamagnetic content of the renal tissue [13], probably due to excess deposition of collagen, which is strongly diamagnetic [31]. Considering the standard deviation, the susceptibility of healthy renal tissue fluctuates around 0 in the current study. In our study, the fibrotic kidney showed a strong diamagnetic susceptibility value, which was substantially lower than that in the healthy renal tissue. Future research in a larger patient cohort is needed to assess the exact diagnostic value of the renal QSM.

There are several limitations of our work. First, the number of healthy volunteers was low and only one patient was included in this study. However, our focus was to develop a robust acquisition protocol and post-processing pipeline for performing in vivo kidney QSM on a clinical MRI system. Second, SPURS and a T2* IDEAL approach were used to remove the unwanted chemical shift effect between water and fat and to calculate the fat-corrected field maps. No fat suppression technique was used in this study, as it would prolong the acquisition time. To address the issue, faster imaging methods such as the volumetric interpolated breath-hold examination (VIBE) [32] or radial acquisition schemes [33] should be taken into consideration when measuring QSM in the abdomen. Third, the generated masks only removed air outside of the abdomen. The air in the intestinal tract and lungs was still present in the susceptibility calculations. This could lead to non-local errors in the local fields across the ROI [34]. However, we showed good intrasubject reproducibility in the current study, suggesting that the error due to air inside the abdomen is neglectable. Furthermore, the resolution of the MR images was rather low, which might have led to susceptibility underestimation [35, 36]. To overcome this limitation, the raw data were zero-filled prior to the QSM post-processing. Besides, the QSM data were acquired in a single breath-hold of 33 s, which might be difficult to perform for sick and elderly subjects. A further optimization of the multi-echo 3D gradient echo sequence is, therefore, needed to reduce the scan time while maintaining the image quality.

In conclusion, the feasibility of in vivo QSM in the human kidney was successfully demonstrated in the current study with good reproducibility. The obvious difference of the QSM values between healthy kidneys and a fibrotic kidney indicates the possible diagnostic potential of QSM. Further studies with larger patient populations should be performed to prove the diagnostic relevance of QSM for functional renal MR imaging.

## Electronic supplementary material

Below is the link to the electronic supplementary material.

Supplementary file1 (TIFF 3332 KB)

Supplementary file2 (TIFF 3368 KB)

Supplementary file3 (TIFF 1771 KB)

Supplementary file4 (DOCX 14 KB)

## References

[1] Mendichovszky I, Pullens P, Dekkers I, Nery F, Bane O, Pohlmann A, de Boer A, Ljimani A, Odudu A, Buchanan C, Sharma K, Laustsen C, Harteveld A, Golay X, Pedrosa I, Alsop D, Fain S, Caroli A, Prasad P, Francis S, Sigmund E, Fernández-Seara M, Sourbron S (2020). Technical recommendations for clinical translation of renal MRI: a consensus project of the Cooperation in Science and Technology Action PARENCHIMA. Magn Reson Mater Phy Biol Med.

[2] Selby NM, Blankestijn PJ, Boor P, Combe C, Eckardt KU, Eikefjord E, Garcia-Fernandez N, Golay X, Gordon I, Grenier N, Hockings PD, Jensen JD, Joles JA, Kalra PA, Krämer BK, Mark PB, Mendichovszky IA, Nikolic O, Odudu A, Ong ACM, Ortiz A, Pruijm M, Remuzzi G, Rørvik J, de Seigneux S, Simms RJ, Slatinska J, Summers P, Taal MW, Thoeny HC, Vallée JP, Wolf M, Caroli A, Sourbron S (2018). Magnetic resonance imaging biomarkers for chronic kidney disease: a position paper from the European Cooperation in Science and Technology Action PARENCHIMA. Nephrol Dial Transplant.

[3] Caroli A, Pruijm M, Burnier M, Selby NM (2018). Functional magnetic resonance imaging of the kidneys: where do we stand? The perspective of the European COST Action PARENCHIMA. Nephrol Dial Transplant.

[4] Kretzler M, Cohen CD, Doran P, Henger A, Madden S, Gröne EF, Nelson PJ, Schlöndorff D, Gröne HJ (2002). Repuncturing the renal biopsy: strategies for molecular diagnosis in nephrology. J Am Soc Nephrol.

[5] Corwin HL, Schwartz MM, Lewis EJ (1988). The importance of sample size in the interpretation of the renal biopsy. Am J Nephrol.

[6] Haacke EM, Liu S, Buch S, Zheng W, Wu D, Ye Y (2015). Quantitative susceptibility mapping: current status and future directions. Magn Reson Imaging.

[7] Liu C, Wei H, Gong N-J, Cronin M, Dibb R, Decker K (2015). Quantitative susceptibility mapping: contrast mechanisms and clinical applications. Tomography.

[8] Schweser F, Deistung A, Reichenbach JR (2016). Foundations of MRI phase imaging and processing for quantitative susceptibility mapping (QSM). Z Med Phys.

[9] Li W, Wu B, Liu C (2011). Quantitative susceptibility mapping of human brain reflects spatial variation in tissue composition. NeuroImage.

[10] Wang Y, Liu T (2015). Quantitative susceptibility mapping (QSM): decoding MRI data for a tissue magnetic biomarker. Magn Reson Med.

[11] Duyn JH, Van Gelderen P, Li TQ, De Zwart JA, Koretsky AP, Fukunaga M (2007). High-field MRI of brain cortical substructure based on signal phase. Proc Natl Acad Sci USA.

[12] Liu C (2010). Susceptibility tensor imaging. Magn Reson Med.

[13] Xie L, Sparks MA, Li W, Qi Y, Liu C, Coffman TM, Johnson GA (2013). Quantitative susceptibility mapping of kidney inflammation and fibrosis in type 1 angiotensin receptor-deficient mice. NMR Biomed.

[14] Zivadinov R, Tavazzi E, Bergsland N, Hagemeier J, Lin F, Dwyer MG, Carl E, Kolb C, Hojnacki D, Ramasamy D, Durfee J, Weinstock-Guttman B, Schweser F (2018). Brain iron at quantitative MRI is associated with disability in multiple sclerosis. Radiology.

[15] Li DTH, Hui ES, Chan Q, Yao N, Chua SE, McAlonan GM, Pang SYY, Ho SL, Mak HKF (2018). Quantitative susceptibility mapping as an indicator of subcortical and limbic iron abnormality in Parkinson’s disease with dementia. NeuroImage Clin.

[16] Sun H, Klahr AC, Kate M, Gioia LC, Emery DJ, Butcher KS, Wilman AH (2018). Quantitative susceptibility mapping for following intracranial hemorrhage. Radiology.

[17] Sharma SD, Hernando D, Horng DE, Reeder SB (2015). Quantitative susceptibility mapping in the abdomen as an imaging biomarker of hepatic iron overload. Magn Reson Med.

[18] Xie L, Layton AT, Wang N, Larson PEZ, Zhang JL, Lee VS, Liu C, Johnson GA (2016). Dynamic contrast-enhanced quantitative susceptibility mapping with ultrashort echo time MRI for evaluating renal function. Am J Physiol Ren Physiol.

[19] Xie L, Dibb R, Cofer GP, Li W, Nicholls PJ, Johnson GA, Liu C (2015). Susceptibility tensor imaging of the kidney and its microstructural underpinnings. Magn Reson Med.

[20] Dong J, Liu T, Chen F, Zhou D, Dimov A, Raj A, Cheng Q, Spincemaille P, Wang Y (2015). Simultaneous phase unwrapping and removal of chemical shift (SPURS) using graph cuts: application in quantitative susceptibility mapping. IEEE Trans Med Imaging.

[21] Li W, Wang N, Yu F, Han H, Cao W, Romero R, Tantiwongkosi B, Duong TQ, Liu C (2015). A method for estimating and removing streaking artifacts in quantitative susceptibility mapping. NeuroImage.

[22] Bechler E, Stabinska J, Wittsack H (2019). Analysis of different phase unwrapping methods to optimize quantitative susceptibility mapping in the abdomen. Magn Reson Med.

[23] Hernando D, Kramer JH, Reeder SB (2013). Multipeak fat-corrected complex R2* relaxometry: theory, optimization, and clinical validation. Magn Reson Med.

[24] Zhou D, Liu T, Spincemaille P, Wang Y (2014). Background field removal by solving the Laplacian boundary value problem. NMR Biomed.

[25] Liu J, Liu T, de Rochefort L, Ledoux J, Khalidov I, Chen W, Tsiouris AJ, Wisnieff C, Spincemaille P, Prince MR, Wang Y (2012). Morphology enabled dipole inversion for quantitative susceptibility mapping using structural consistency between the magnitude image and the susceptibility map. NeuroImage.

[26] Wei H, Dibb R, Zhou Y, Sun Y, Xu J, Wang N, Liu C (2015). Streaking artifact reduction for quantitative susceptibility mapping of sources with large dynamic range. NMR Biomed.

[27] Lin H, Wei H, He N, Fu C, Cheng S, Shen J, Wang B, Yan X, Liu C, Yan F (2018). Quantitative susceptibility mapping in combination with water–fat separation for simultaneous liver iron and fat fraction quantification. Eur Radiol.

[28] Kido A, Kataoka M, Yamamoto A, Nakamoto Y, Umeoka S, Koyama T, Maetani Y, Isoda H, Tamai K, Morisawa N, Saga T, Mori S, Togashi K (2010). Diffusion tensor MRI of the kidney at 3.0 and 1.5 Tesla. Acta Radiol.

[29] Jafari R, Sheth S, Spincemaille P, Nguyen TD, Prince MR, Wen Y, Guo Y, Deh K, Liu Z, Margolis D, Brittenham GM, Kierans AS, Wang Y (2019). Rapid automated liver quantitative susceptibility mapping. J Magn Reson Imaging.

[30] Li J, Lin H, Liu T, Zhang Z, Prince MR, Gillen K, Yan X, Song Q, Hua T, Zhao X, Zhang M, Zhao Y, Li G, Tang G, Yang G, Brittenham GM, Wang Y (2018). Quantitative susceptibility mapping (QSM) minimizes interference from cellular pathology in R2* estimation of liver iron concentration. J Magn Reson Imaging.

[31] Luo J, He X, d’Avignon DA, Ackerman JJH, Yablonskiy DA (2010). Protein-induced water 1H MR frequency shifts: contributions from magnetic susceptibility and exchange effects. J Magn Reson.

[32] Rofsky NM, Lee VS, Laub G, Pollack MA, Krinsky GA, Thomasson D, Ambrosino MM, Weinreb JC (1999). Abdominal MR imaging with a volumetric interpolated breath-hold examination. Radiology.

[33] Yedururi S, Kang HC, Wei W, Wagner-Bartak NA, Marcal LP, Stafford RJ, Willis BJ, Szklaruk J (2016). Free-breathing radial volumetric interpolated breath-hold examination vs breath-hold cartesian volumetric interpolated breath-hold examination magnetic resonance imaging of the liver at 1.5 T. World J Radiol.

[34] Schweser F, Robinson S, de Rochefort L, Li W, Bredies K (2017). An illustrated comparison of processing methods for phase MRI and QSM: removal of background field contributions from sources outside the region of interest. NMR Biomed.

[35] Karsa A, Punwani S, Shmueli K (2019). The effect of low resolution and coverage on the accuracy of susceptibility mapping. Magn Reson Med.

[36] Zhou D, Cho J, Zhang J, Spincemaille P, Wang Y (2017). Susceptibility underestimation in a high-susceptibility phantom: dependence on imaging resolution, magnitude contrast, and other parameters. Magn Reson Med.

