# Correction to: Feasibility of quantitative susceptibility mapping (QSM) of the human kidney

**DOI:** 10.1007/s10334-021-00960-x

**Published:** 2021-10-13

**Authors:** Eric Bechler, Julia Stabinska, Thomas Thiel, Jonas Jasse, Romans Zukovs, Birte Valentin, HansJörg Wittsack, Alexandra Ljimani

**Affiliations:** 1 Department of Diagnostic and Interventional Radiology, Medical Faculty, Heinrich Heine University Düsseldorf, Moorenstr. 5, 40225 Düsseldorf, Germany; 2 Department of Haematology, Oncology and Clinical Immunology, Medical Faculty, Heinrich Heine University Düsseldorf, Düsseldorf, Germany

## Correction to: Magnetic Resonance Materials in Physics, Biology and Medicine (2021) 34:389–397 10.1007/s10334-020-00895-9

The article Feasibility of quantitative susceptibility mapping (QSM) of the human kidney, written by Eric Bechler, Julia Stabinska, Thomas Thiel, Jonas Jasse, Romans Zukovs, Birte Valentin, Hans‑Jorg Wittsack and Alexandra Ljimani, was originally published Online First without Open Access. After publication in volume 34, issue 3, page 389–397 the author decided to opt for Open Choice and to make the article an Open Access publication. Therefore, the copyright of the article has been changed to © The Author(s) 2021 and this article is licensed under a Creative Commons Attribution 4.0 International License, which permits use, sharing, adaptation, distribution and reproduction in any medium or format, as long as you give appropriate credit to the original author(s) and the source, provide a link to the Creative Commons licence, and indicate if changes were made. The images or other third party material in this article are included in the article’s Creative Commons licence, unless indicated otherwise in a credit line to the material. If material is not included in the article’s Creative Commons licence and your intended use is not permitted by statutory regulation or exceeds the permitted use, you will need to obtain permission directly from the copyright holder. To view a copy of this licence, visit http://creativecommons.org/licenses/by/4.0/.

Open Access funding enabled and organized by Projekt DEAL.

The original article has been corrected.

