# Supplementary figures and images for: Feasibility of quantitative susceptibility mapping (QSM) of the human kidney
Source: MAGMA. 2020 Nov 24;34(3):389–97. doi: 10.1007/s10334-020-00895-9 (PMC8492554; doi:10.1007/s10334-020-00895-9)

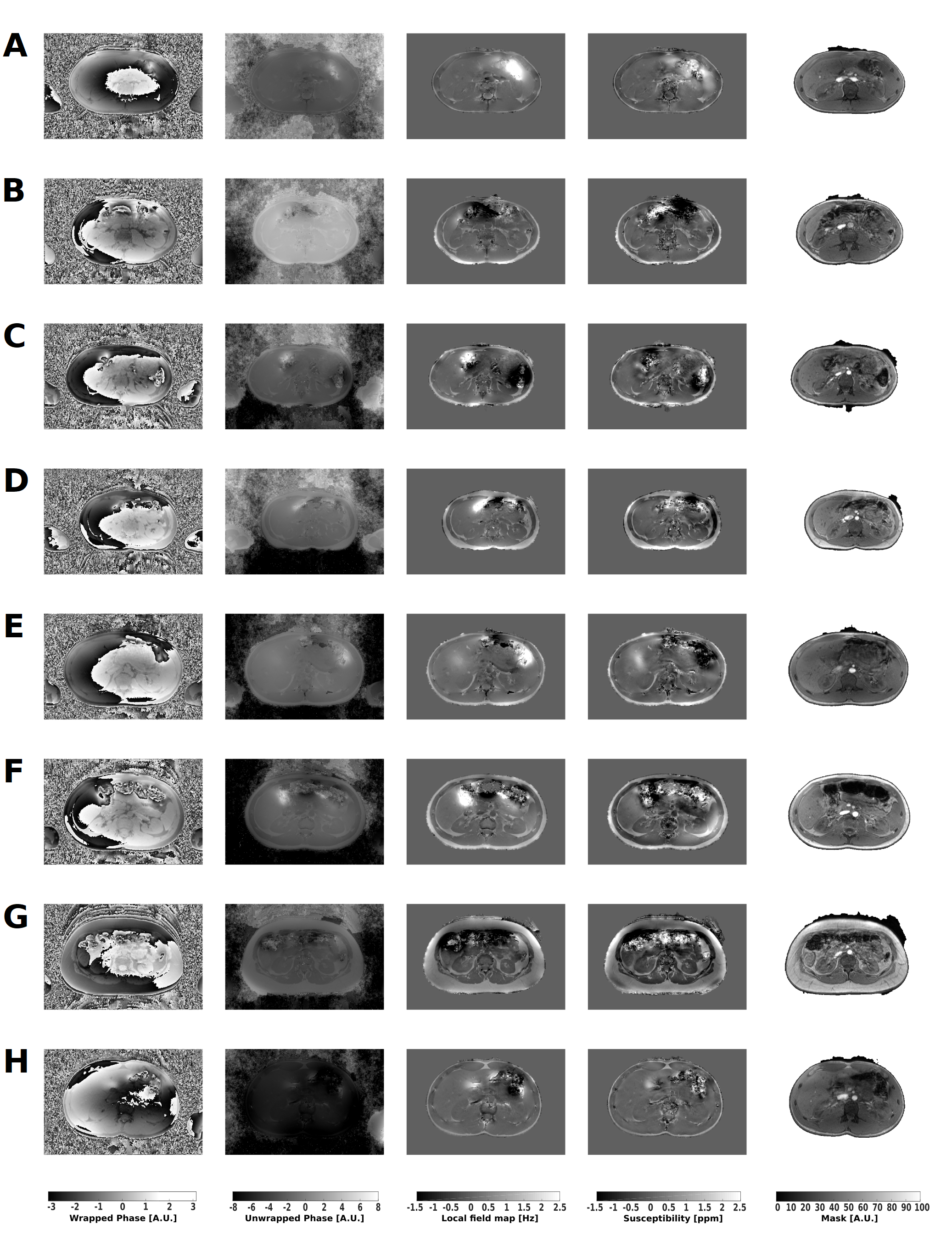

Supplement: Supplementary file 1 — Supplementary file1 (TIFF 3332 KB) [file 10334_2020_895_MOESM1_ESM.tiff]

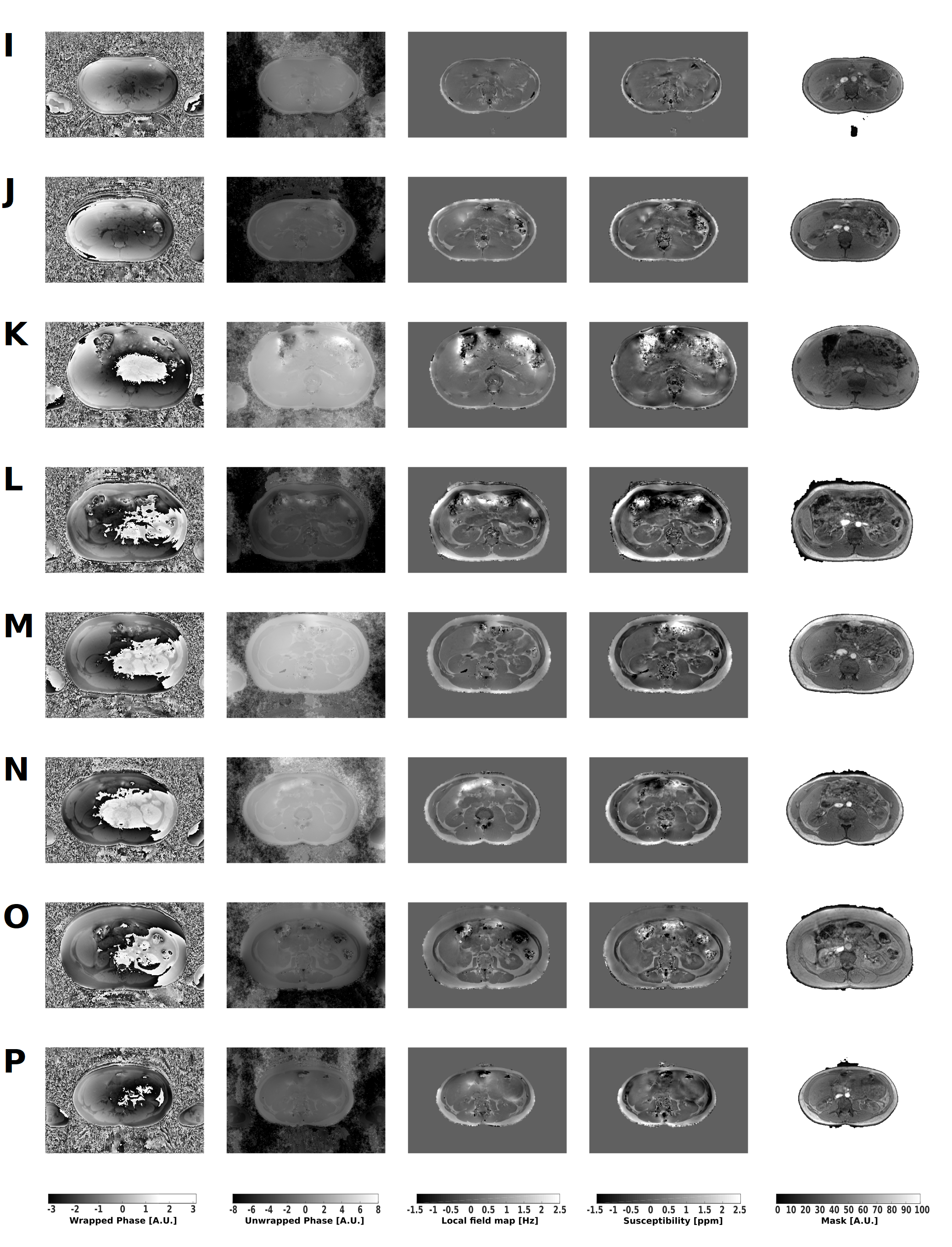

Supplement: Supplementary file 2 — Supplementary file2 (TIFF 3368 KB) [file 10334_2020_895_MOESM2_ESM.tiff]

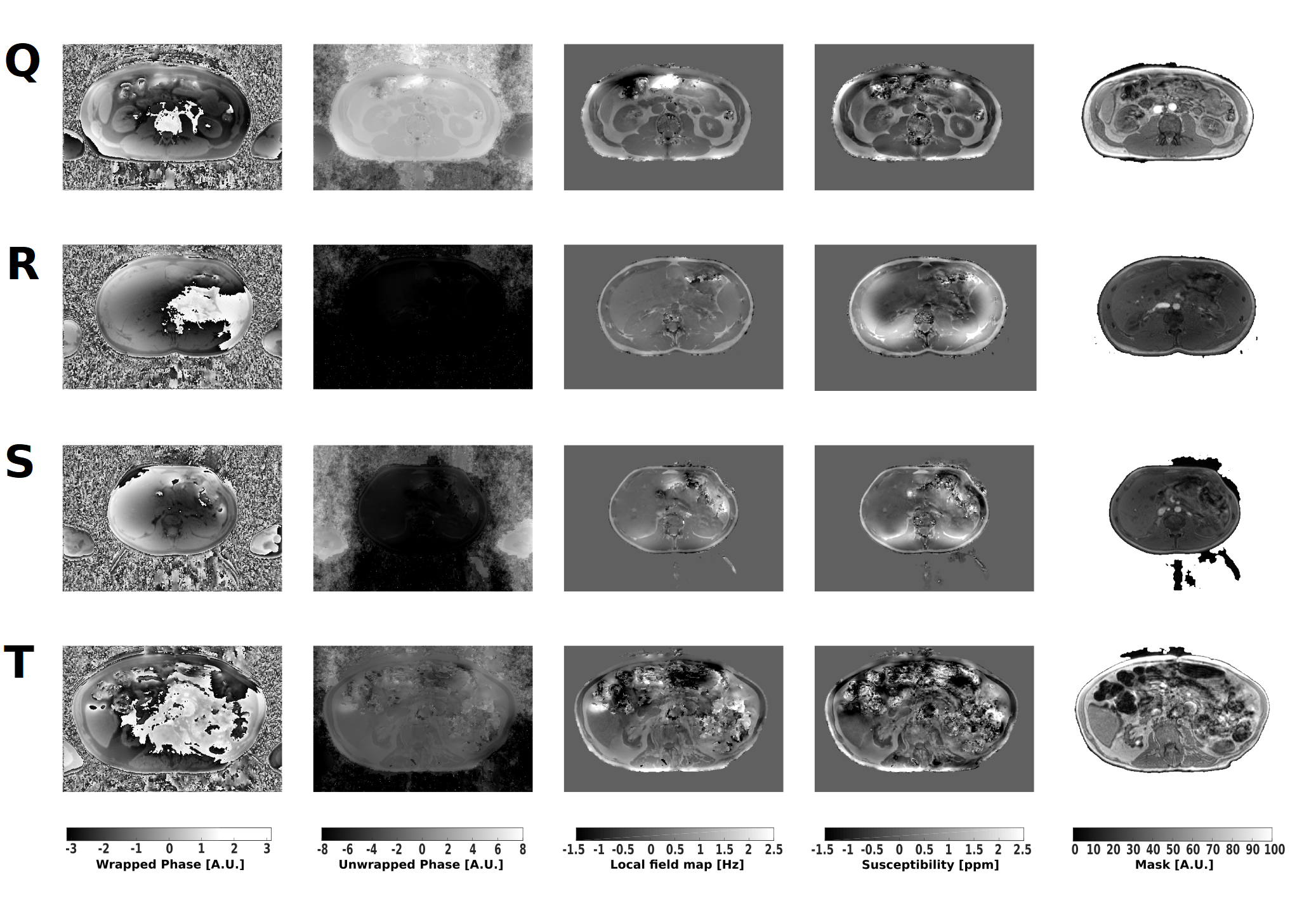

Supplement: Supplementary file 3 — Supplementary file3 (TIFF 1771 KB) [file 10334_2020_895_MOESM3_ESM.tiff]
