# Supplementary material for: Feasibility of quantitative susceptibility mapping (QSM) of the human kidney
Source: MAGMA. 2020 Nov 24;34(3):389–97. doi: 10.1007/s10334-020-00895-9 (PMC8492554; doi:10.1007/s10334-020-00895-9)
Supplement: Supplementary file 4 — Supplementary file4 (DOCX 14 KB) [file 10334_2020_895_MOESM4_ESM.docx]

**Supplement material**

**Fig. S1-S3: Individual images of wrapped and unwrapped phase as well as local field and susceptibility maps and the corresponding mask** of the upper abdomen of all subjects included in the study.

| **Subject** | **Susceptibility (ppm)** |
| --- | --- |
| 1 | -0.135 ± 0.002 |
| 2 | -0.133 ± 0.003 |
| 3 | -0.130 ± 0.001 |
| 4 | -0.184 ± 0.001 |
| 5 | -0.183 ± 0.003 |
| 6 | -0.416 ± 0.002 |
| 7 | -0.520 ± 0.006 |
| 8 | -0.005 ± 0.004 |
| 9 | -0.087 ± 0.003 |
| 10 | -0.172 ± 0.001 |
| 11 | -0.300 ± 0.002 |
| 12 | -0.202 ± 0.002 |
| 13 | -0.388 ± 0.001 |
| 14 | -0.134 ± 0.001 |
| 15 | -0.147 ± 0.008 |
| 16 | -0.180 ± 0.005 |
| 17 | -0.255 ± 0.002 |
| Patient | -0.256 ± 0.006 |

**Table S1: Average susceptibility values of the paravertebral muscle tissue** of the 17 healthy volunteers and the patient with renal fibrosis. These values were used as reference for further QSM quantification.
